# Educational intervention for the prevention of occupational neck pain: protocol of randomized trial

**DOI:** 10.1186/s13063-022-06247-3

**Published:** 2022-04-08

**Authors:** Zohreh Moradi, Sedigheh Sadat Tavafian, Seyedeh Somayeh Kazemi

**Affiliations:** 1 Department of Health Education and Health Promotion, Faculty of Medical Sciences, Tarbiat Modares University, Tehran, Iran; 2 Chalous Health Network, Mazandaran University of Medical Sciences, Chalous, Iran

**Keywords:** Work-related neck pain, Social media, Teacher, Neck pain

## Abstract

**Background:**

Neck pain is one of the most common work-related musculoskeletal disorders (WMSDs). It has important social and economic consequences such as reduced productivity due to absenteeism, leave, and early retirement and financial losses due to medical expenses for the workforce especially teachers. This study aims to evaluate whether a model-based social media intervention could change the high-risk behaviors that cause work-related neck pain among teachers.

**Methods:**

This is a randomized controlled trial that will be done in three steps. The first stage is a qualitative study to obtain the items and areas of the researcher-made questionnaire based on the health belief model (HBM), the second stage is the psychometric evaluation of the questionnaire, and the third stage is designing and implementation of model-based educational intervention in social media context. The study population is teachers who working in junior high school in the 19th district of education minister in Tehran, Iran, which are randomly divided into two groups of intervention and control. The intervention group receives training packages on social media, and the control group does not receive any training. The educational intervention tries to improve the knowledge, attitude, skills, and self-efficacy in adopting neck pain prevention behaviors among teachers. The study will also assess whether the intervention can promote preventive neck pain behavior among teachers.

**Discussion:**

Work-related neck pain can have a negative impact on teachers’ health. This study is an attempt to investigate the impact of developed interventions in promoting preventive behavior regarding work-related neck pain through social media context.

**Trial registration:**

Iranian registry of Clinical Trial (IRCT) IRCT20210301050542N1. Registered on 16 March 2021

Ethics code: IR.MODARES.REC.1399.163

## Background

Work-related musculoskeletal disorders (WMSDs) are one of the largest occupational health problems in the working population in developing and developed countries, with the highest number of occupational complaints with different severity [1, 2]. Furthermore, social and economic consequences for the workforces have been evident in many studies [3, 4]. Performing work tasks in inappropriate physical, psychological, and ergonomic conditions causes disorders in the bones, nerves, tendons, ligaments, muscles, and blood vessels and causes pain in the neck, back, shoulders, elbows, wrists, arms, and hands [5–7]. It has been estimated that about 44% of all occupational diseases are musculoskeletal disorders which caused by WMSDs [8].

Neck pain is one of the most common and costly health challenges in the workplace [9, 10] that affects about 52–58% of the population during life [8]. Neck pain (NP) has a significant impact on social functioning and health status which lead to inability to perform daily activities and consequently reduced quality of life as well as imposing health costs on society and governments [11, 12]. The prevalence of neck pain varies in different professions [1, 13]. Teachers have been shown to have a higher percentage of work-related musculoskeletal disorders than other occupations [1, 2, 6]; hereby, it has been argued that neck pain among teachers is the fourth their health problem [3, 14].

Neck pain in teachers is caused by several reasons such as age, gender, duration of employment, use of inappropriate body postures, uncontrolled stress, high workload, prolonged sitting and standing, and job dissatisfaction as well as doing monotonous and repetitive tasks [4, 15–17]. The consequences of neck pain on teachers’ life include frequent leave and absences from workplace, reduced individual and social functioning, early retirement, inability to perform activities, and the imposition of health costs [18].

It has been discussed that various factors such as demographic factors (body mass index, vitamin D deficiency), physical factors such as excessive computer use, prolonged sitting and standing, excessive bending of the trunk, squatting, bending over neck forward backward, keeping hands above shoulder level for a long time, doing strenuous activities and unprincipled exercise at work or outside, lack of adequate rest time during working hours and psychosocial factors such as work-related stress, lack of attention and support from colleagues, marital and family relationships, job dissatisfaction, weak interpersonal relationships, organizational characteristics, and financial–social aspects cause neck pain [19–21]. Many of these mentioned various factors is rooted in unhealthy and high-risk behaviors which done by individuals specially teachers [11, 12, 22, 23]. Reducing neck pain was possible if teachers could observe and maintain correct behaviors during their working and daily activities [7, 24]. It seems that the main obstacles in training teachers to do preventive neck pain behaviors are lack of time required for on-the-job training, lack of ergonomic equipment in the workplace, lack of adequate rest time at work, and health care costs [22].

Education through social media or participatory internet refers to a set of activities and communications that are done using electronic devices such as audio/video equipment, computer networks, and virtual tools on the Internet. In fact, all communication that is done through the internet and in the context of social networks and remotely without face-to-face interaction and leads to learning is called internet learning. In fact, this type of communication can provide a fraction of different information to a large number of users over long distances which is also economically viable [25, 26]. At present, the use of social networks has become as one of the best ways to promote health due to its effectiveness in reaching the general audience [27]. Moreover, due to the outbreak of the massive corona pandemic, it will be impossible to implement in- person educational intervention for teachers, so social interactive educational intervention is really recommended.

**Fig. 1:**
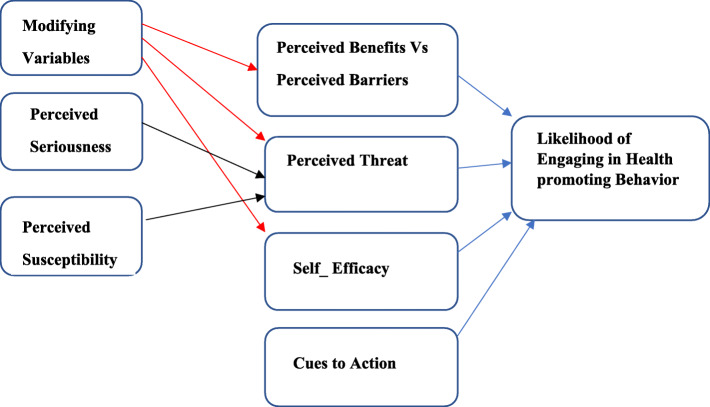
The constructs of health belief model

Different studies showed that model-based interventions are more effective to reducing musculoskeletal disorders compared with non-model ones [28, 29]. One of the best effective models that address lacked perception of people to promote preventive behaviors is the health belief model (HBM); its constructs were shown in Fig. 1 [28, 30, 31]. Numerous studies have shown that educational interventions based on health belief model is so effective on promoting preventive behaviors and behavior modification which could reduce musculoskeletal disorders such as low back pain and neck and shoulder pain [32–34].

Accordingly, the first phase of this research project was done through interviews with the teachers based on this model, and the results clarified that they did not perceive the severity of neck pain consequences. Furthermore, they did not consider themselves as susceptible individuals for neck pain if they would not comply with preventive behaviors. On the other hand, they did not understand the benefits of neck pain preventive behaviors and so they mentioned many barriers including being unskilled to do these preventive behaviors. In other words, they lacked sufficient self-efficacy. Therefore, it was concluded that health belief model would be a proper model which possibly can overcome these causative factors of not doing preventive behaviors by teachers.

Therefore, according to address these limitations, this protocol aims to develop an intervention based on the health belief model in social media context in order to promote preventive behaviors among the school teachers.

*To achieve the overall goal, three following phases needed to be considered:*

Phase1: Carrying out a qualitative study to determine the leading factors influence doing neck pain preventive behaviors and domain/item generation of researcher-made questionnaire

Phase 2: Psychometrics of the developed questionnaire

Phase 3: Designing and implementation of an educational intervention based on the health belief model in the context of social networks to adopt behaviors that to reduce neck pain in teachers

## Methods/design

The design of this protocol is a randomized clinical trial. The final purpose is to develop and evaluate a model-based social media intervention regarding behavioral work-related neck pain among school teachers. To reach this aim, the following phases which are shown in Table 1 should be done.

**Table 1:** The study overview

Phases and participants	Aim	Methods
Phase 1		
Qualitative study on teachers	Step 1: Identifying occupational factors that cause neck pain, impact of neck pain on daily life activities, neck pain preventive behaviors, and affective factors	In-depth interviews
Researcher	Step 2: Designing of an initial questionnaire	Based on step 1
Phase 2		
Psychometrics evaluation	To validate the questionnaire Preparation of the final questionnaire	By research team factor analysis, varimax questionnaire, rotation, content validity index, and impact score
Phase 3		
Design interventions researcher	Step 1: Intervention group training	Based on social media
Implementation teachers	Step 2: Transfer educational content to intervention group	Based on social media
Intervention evaluation teachers	Step 3: Evaluate the program Identify the impact of the program in the intervention group	Questionnaire checklist

The first phase of the research is a qualitative study that is done through interviewing with 25 teachers. Criteria for entering into this phase of the research are as follows: having a work-related neck pain experience and willingness and ability to share his/her experiences. Data collection will be done through semi-structured interview guild line with the participants. The questions are designed based on the health belief model. Participants are asked questions about their experiences about work-related neck pain, its consequences and effects on their work and life, the extent to which teachers suffer from neck pain, the behavioral factors that cause this problem, and the effective neck pain preventive behaviors as well as the benefits/barriers of doing the behaviors and how they are confident that they could do these behaviors. Based on the content analysis of the collected data from the interview, the main categories based on HBM and items of each domain of the researcher-made questionnaire are determined.

The second phase of this study is psychometry evaluation of the researcher-made questionnaire that is made in previous phase. In this phase, the researcher-made questionnaire is subject to content validity and face validity by 15 specialists in health education/health promotion, ergonomics, physiotherapy, and occupational health. Exploratory factor analysis and scale correlation matrix will also be used to evaluate the construct validity and to obtain the final items of the questionnaire in each domain.

**Fig. 2:**
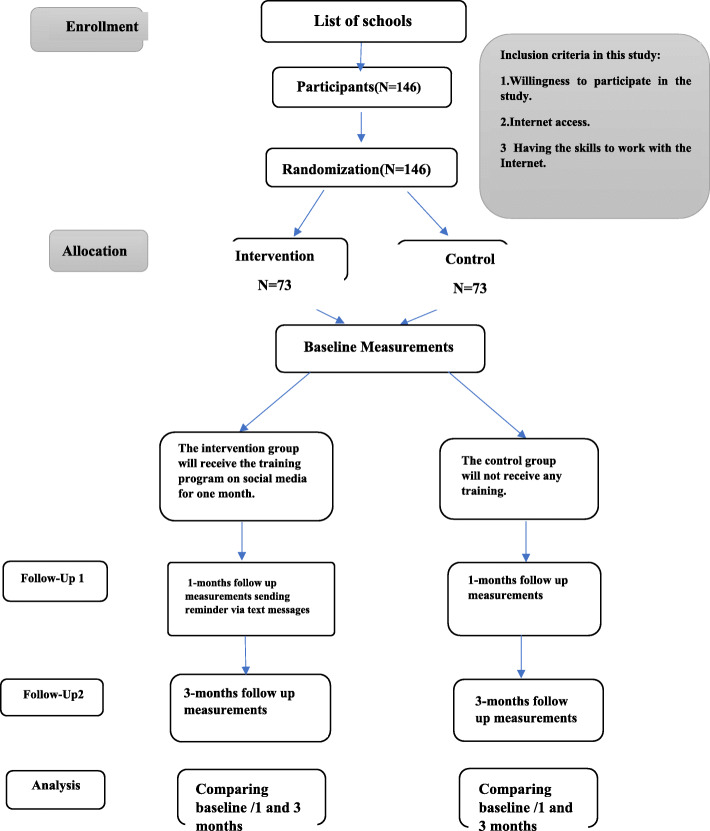
Consort flow diagram

The third phase of the study includes designing, implementing and evaluation of a model -based social media intervention for promoting preventive behaviors due to work-related neck pain (Fig. 2).

### Sampling

At this stage, a list of all 26 schools in district 19 of education ministry in Tehran will be prepared. From the schools, people are randomly selected and then randomly divided into two groups of intervention and control. The intervention group receives a training program while the control group does not receive any training program. Participants are evaluated in three time points of before the intervention, 1 and 3 months after the intervention. The study setting will be public schools in the 19th district of education ministry in Tehran city of Iran. The schools in this district are located in center of city that is almost similar to majority of other schools. Participants are teachers of junior high school.

### Calculating sample size

Based on the similar existed evidence (21) and considering the 95% confidence level and 80% test power, the number of necessary samples for each group (intervention and control) according to Pocock formula is 63 people which plus 10% for dropout would be of 73 people in each group finally. For randomization, first, a list of teachers’ telephone numbers with a specific code is prepared, and the codes are poured into a bag and randomly divided into two groups of intervention and control. This study is a double-blind study, so the researcher and participants are not aware of about the grouping of individuals.

### Consent or assent

The informed consent form is completed online by all study participants.

### Inclusion and exclusion criteria

The inclusion criteria in this study are as follows: willingness to participate in the study, access to the internet, and having the skills to use the internet. The exclusion criteria include the following: having a second job, suffering from congenital musculoskeletal disorders, history of surgery in the neck region, history of neck vertebral fractures and pathologic neck pain, and medical prohibition in doing neck preventive behaviors.

The main purpose of this study is to promote preventive neck pain behaviors, so the first phase of the study identifies the behavioral factors that cause neck pain to address them through designing and implementing proper educational intervention based on the health belief model. The findings of this stage are obtained through semi-structured interviews based on the health belief model which clarify the behavioral factors affecting neck pain. Based on HBM, these factors might be included perceived sensitivity, perceived severity, perceived benefits, perceived barriers, cues to action, and self-efficacy [34].

### Designing and development of educational intervention

#### Educational intervention content

Definition of occupational neck pain and its causes, benefits of healthy preventive behaviors, strategies to deal with barriers towards doing neck pain prevention behaviors, improving self-efficacy in adopting and performing neck pain preventive behaviors, stressors in the workplace and their effects on neck pain preventive behaviors, stress management techniques, effective communication, angry management skills, excitement management skills, ergonomic principles on neck pain prevention, correct movements that reduce neck pain, stretching movements that strengthen neck muscles, sleeping properly, proper sitting and standing, correct posture while working with computer/mobile phone, and all skills regarding doing all daily activities in correct posture are included in intervention content. Before implementing intervention, the prepared educational contents are reviewed by experts in the fields of health education/health promotion, occupational health, ergonomics, and physiotherapy and also psychology in terms of scientific and appropriateness validity. Moreover, before presenting the educational contents to the participants in intervention group, the contents are examined in terms of simplicity, clarity, comprehensibility and appropriateness by a sample of teachers who are similar to target group of the study. Moreover, accuracy of their mobile device and internet access will be checked during educational intervention transferring.

#### Implementation of intervention

Participants can communicate with the researcher by email and phone call. The intervention group will receive training in the form of webinars, group discussions, questions and answers, videos, animations, posters, pamphlets, and infographics. The procedure of teacher training is supervised by the researcher. The training will be provided to the intervention group through social media for 1 month. After, the reminder training will be provided to the intervention group for 3 months. During this period, the control group will not receive any intervention. However, the training package will be provided to the control group after the study is finished. Although there is no auditing plan in this study, if there are any participants with this problem, he/she will be excluded from the study.

#### Questionnaire used in this study

The main outcome of the study is promoting of neck pain preventive behaviors that will be assessed using a researcher-made questionnaire. Data collection instrument is a researcher-made questionnaire that is completed and collected in three stages. Therefore, before the intervention, 1 month after the intervention and 3 months after the intervention, the questionnaire is presented to the intervention and control groups for completion. The data obtained in three stages in both groups are analyzed to determine whether the educational intervention is effective in promoting neck pain preventive  behaviors in teachers.

#### Scoring questionnaire

Questionnaires are completed anonymously to gain participants’ trust in the intervention. This questionnaire has different domains of HBM such as knowledge, perceived sensitivity, perceived severity, perceived barriers, perceived benefits, and cues to action, self-efficacy, and behavior. Knowledge questions have the Likert form with three-part spectrum as true (with score 2), no idea (with score 1), wrong (with score 0), and domain questions of perceived sensitivity, perceived intensity, perceived barriers, perceived benefits, self-efficacy, and cues to action which have 5-part Likert spectrum form like completely agree with score 5, agree with score 4, no idea with score 3, disagree with score 2, and completely disagree with score 1. Behavior questions are considered as a five-part Likert scale: never with score 1, rarely with score 2, sometimes with score 3, often with score 4, and always with score 5. This questionnaire measures the variables of knowledge, perceived threats, self-efficacy, reinforcing factor, and barriers towards behavior doing. A higher score in each area of the questionnaire indicates a better situation.

#### Public participation

In this study, public participation of the participants was used to design an educational social media intervention and evaluate its impact on promoting preventive behaviors.

### Statistical analysis

#### Data management

The obtained data is analyzed in three stages.

Phase 1: Analysis of data from a qualitative study to determine potential domains and items of the researcher-made questionnaire

Phase 2: Factor analysis of the questions to determine the definitive domains and items of the researcher-made questionnaire

Phase 3: Analysis of data obtained from both groups of intervention and control at three time points of before intervention, 1-month and 3-month follow-ups

#### Data monitoring

The data obtained from the qualitative study is analyzed based on Graneheim and Landman algorithms in qualitative content analysis. The MAXQDA software will be used to encode semi-structured interview data.

To evaluate the validity of the structure, factor analysis and scale correlation matrix and the Kaiser_Meyer_Olkin (KMO) Index as well as Bartlett’s test sphericity will be used. The factor structure of the questionnaire is extracted using varimax rotation.

The quantitative data in clinical trial will be analyzed using SPSS V24. Descriptive statistics will include frequencies, means, and standard deviations. The Kolmogorov-Smirnov test will be used to check the normal distribution of data. Moreover, to compare the means between the two intervention and control groups, *T* test is used. Paired *t*-test will be used to compare the means in each group at two points in time, and ANOVA test will be used to compare the means in each group at three points in time. To analyze and evaluate the correlation between demographic variables, correlation tests (Pearson for parametric data and Spearman or Kendall for non-parametric data) and chi-square test will be used.

## Discussion

One of the features of this study is paying attention to promoting health in the workplace. This study will investigate the effect of interventions in promoting preventive behaviors and reducing occupational neck pain among teachers. The main framework of this study will be designing a model-based intervention in social media context to prevent teachers’ neck pain.

The strengths of this study include conducting a qualitative study to design a researcher-made questionnaire, psychometric phase of the questionnaire, designing an educational intervention based on HBM, and implementing the specific intervention using social media and finally compare it with the control group to determine its effects. However, if this intervention is cost-effective, its’ flexibility and accessibility makes it easy for users to access educational content easily. One of the weakness points of the study is the self-report procedures of people about their neck pain perception and behaviors. Another limitation of the study is the lack of time for collecting data on 6- and 12-month follow-ups to determine its continued maintained effects which should be considered in future studies.

## Ethics and dissemination

1. Obtaining a license from the Faculty of Medical Sciences of Tarbiat Modares University with ID code (IR.MODARES.REC.1399.163)

2. Registration in Iran Clinical Trial Center with ID code (IRCT20210301050542 N1)

3. Obtaining a letter of introduction from of Tarbiat Modares University for the Ministry of Education in Tehran

4. Obtaining a license from the Education Organization of District 19 of Tehran

5. Obtaining informed consent from the participants in the study by the researcher

6. No need to the write first and last name on the questionnaires

7. Assuring participants that their information is confidential

8. Providing educational content to the control group after the end of the research

### Dissemination policy

The publishing project finding in international journals is welcome. The authors would like to present the program results to teachers’ community and other stakeholders in the Ministry of Education in Iran.

### Ancillary and post-trial care

An ergonomist cooperates with the researchers to do the ancillary and post-trial care in this duty.

### Ethics permission

The Ethics Committee for Health Research of Tarbiat Modares University approved the ethical principles with the following code: IR.MODARES.REC.1399.163. Informed consent form will be obtained from all participants. The data (when ready) will be available from the corresponding author on request.

## Data Availability

The data will be available from the corresponding author on request.
